# Notes and Comments

**Published:** 1923-11

**Authors:** 


### NOTES AND COMMENTS.

### PURE FOOD.

THERE has of late been a good deal of discussion as to the use of preservatives in food and the lack of good domestic methods of food storage. That preservatives are used in far too many cases is as notorious as the evil nature of their results. To say that certain foods would not keep without them is a very poor argument, and we agree with Dr. Howarth, the City of London Medical Officer of Health, that "the use of preservatives should be prohibited in all instances other than where official sanction has been formally granted by the Minister of Health." There are very few Medical Officers who do not think with Dr. Howarth, since most of them have had only too much reason to be suspicious of the consequences of artificially delaying the process of decomposition in food. When food is prevented from "going bad" only by chemical precautions the cumulative effect upon the consumer must inevitably be injurious. Dr. Howarth suggests, as a remedy, the distribution to our houses of "cold" in the same way that gas, water, and electricity are distributed. Put roughly, "cold" means liquid ice, and it is likely enough that the time will come when pipes will bring "cold" straight into our larders. We can carry food from the other side of the world in cold storage in perfect safety, yet in hot, or even warm or muggy, weather we cannot store it in our houses for half a week. Perhaps presently some one will invent a meat safe on the principle of the vacuum flask. Such an invention would go far to solve the difficulty of the safe and cleanly storage of food in the most modest homes, pending the arrival of pipe line "cold." Ice is costly and, save in a few large towns, unattainable

### HUMANE SLAUGHTERING.

WE observe with pleasure that, under the auspices of the Model Abattoir Society, Sir Arthur Newsholme has been lecturing before the Royal Society on "Humane Slaughtering in Relation to Public Health." We are as much bound to avoid cruelty in dealing with animals as in dealing with human beings, but in this case considerations of health enter in to reinforce a self-evident maxim. Those who have read the communications on the subject which we have published during the last few months realise only too clearly that much avoidable suffering is inflicted before and during the slaughter of animals for food. Yet we make painfully slow progress towards an amendment of our ways. The number of model abattoirs is still relatively small, and even a great city like Liverpool is only now preparing plans for their creation. We entertain no doubt whatever that Sir Arthur Newsholme is right when he declares that "the maximum cruelty, nuisance, and demoralisation" are caused by the small private slaughter-houses, which are very difficult to regulate. Only too often they are rather tumble-down buildings, dark and dreary, and reeking with odours the inhalation of which must itself inflict suffering upon animals awaiting their doom. Whoever has visited one of these places will never desire, save as a duty, to visit another. Sir Arthur suggests that so long as they exist killing should take place only on fixed days when the Medical Officer of Health can be represented. This is a useful proposal, since it is of real importance that the flesh of animals fevered by fear should be prevented from reaching our tables. But the only real and effective remedy consists in the complete reform of the archaic cruelty of our methods.

### GOING RATTING.

THE first complete week in November will, by ordinance of the Minister of Agriculture, be "Rat Week." This is understood to be the season when the rat finishes his *villegiatura* and returns to the warm town or the cosy farmstead for the winter. From a roaming vagabond taking, like Molière, what he wants wherever he finds it, he becomes a domesticated creature, living respectably in a snug little household run, or finding a cosy lair in a farm, where he has simply to show his nose to espy food. The rat, no doubt, considers that to hunt him at this season is taking a mean advantage of his habits and customs, but it is all his own fault. If he would only become a "clubbable" creature and abandon his evil habits of carrying disease about with him and devouring the crops, mankind would, very likely, be willing to talk gently of living and letting live. As a consumer of garbage he might, other things being equal, be a useful scavanger, but, unhappily, other things are very unequal, and the august Parliament of this land has issued dread decrees against him. One of these decrees even enacts penalties against those who knowingly harbour him, treating him as though he were an Irish rebel or an escaped convict. Unhappily his guilt is patent and his final impenitence beyond question. Therefore Rats Must Go, to use language once made popular by political dissatisfaction. The Ministry of Agriculture will oblige with a list of "suitable poisons," and local authorities are urged to look after the "infestation" of their sewage farms. It seems a pity that we cannot make a holocaust of rats without at the same time sacrificing the English language. The holocaust is necessary enough, since the rat not only sometimes carries disease, but is busily engaged all the time in eating the malt that lies in the house that Jack built and, indeed, in preventing the barley from ever becoming malt and the wheat from ever becoming bread.

### THE CONQUEST OF LIVERPOOL.

IMPROVED sanitation and the increased efficiency of the medical services are working wonders at Liverpool. The report for 1922 of the Medical Officer of Health, Dr. Hope shows results which can rarely have been matched. During the last fifty years the death-rate has been almost exactly halved, having been reduced from a fraction over 28 per thousand to a rather larger fraction over 14. Deaths from digestive disturbances and diseases of infants have fallen to an even larger extent, while the mortality from infectious diseases shows a diminution of 83 per cent. in fifty-two years. These figures naturally exclude influenza, which, as Dr. Hope points out, remains, with cancer, virtually scatheless. The fact that during the half-century the population of Liverpool has nearly doubled makes these results the more remarkable. The Health Department of the great city on the Mersey has reached a point of efficiency of which it may well be proud—an efficiency which other great towns would do well to take as a model. The conditions of a crowded and cosmopolitan port are often unfavourable from the point of view of health, but Liverpool is showing that even unfavourable conditions can be neutralised by energy and by making the best use of progressive sanitary science.

### PASTEURISATION OF MILK.

THAT valuable organisation, the National Clean Milk Society, is adding to the admirable work it has already accomplished by holding a Conference on Pasteurisation. It will take place in the Council Chamber of the London Guildhall on November 21, and there will be two sessions, one presided over by Lord Astor and the other by Lord Dawson of Penn. Papers will be read and discussions opened by nearly a score of distinguished experts, and we are glad to know that a full report of the proceedings will be published in volume form. The methods and processes of Pasteurisation, the physical, chemical, bacteriological, and bio-chemical changes will all be described and debated, and we may well be thankful that so many busy scientific men have been able to devote their time, energy, and love of research to work likely to be so valuable to the public health. The Conference is, of course, highly technical, but its results must necessarily gradually filter down until the man in the street—and his babies—are in full enjoyment of the results of years of enquiry and experiment. We have to look to the general use of chemically pure milk for a very large reduction of disease and no small improvement in the death rate.

### "THE LANCET."

VERY few newspapers live to celebrate their centenary, though it is probable that the number will be larger in the future than in the past by reason of their wider dissemination and the need for specialist organs. There is no more distinguished member of the latter class than *The Lancet*, which reached its hundredth birthday on October 5. In its long history the admirable journal founded by Thomas Wakley has rendered magnificent service, not only to medicine but to the nation generally. Both were signally benefited when, partly from his editorial chair and partly from his seat in Parliament, he compelled the passing of that Medical Registration Act which so greatly raised the status of the doctor and gave the world assurance of the competence of its healers. To it we owe the first movement for the reform of hospital training, while its efforts for the prevention of the adulteration of food were ultimately productive of legislation which has been of high value to health, notwithstanding that a great deal still remains to be done in that direction. Much as it has accomplished in its first century, we can reasonably expect that it will do still more valuable work in its second.

### POPULARISING HEALTH.

NOWHERE in England is what may be called hospital-and-health enterprise livelier than in Sheffield, where the appropriate authorities are never tired of devising schemes for interesting the citizens in the public health. Last year the Education Department, the Health Committee, the Insurance Committee, the Joint Hospitals' Council, and the Association of Hospital Contributors combined to arrange a series of lectures on "Health Education," which were so successful that they attracted at least ten thousand listeners. The enterprise is now being repeated, and seems likely to become an annual event. Between the beginning of October and the end of March a series of nine lectures will be delivered by distinguished authorities upon such subjects as clothing and its relation to health, the value and limitations of physical exercise, the preventable mortality of middle age, health and ventilation. The nine lecturers include Sir Arthur Newsholme, Dr. Major Greenwood, Professor Pembrey, Dr. Leonard Hill, and Dr. H. M. Vernon, but lest the fare offered should appear to be too solid, it will be diversified by music and singing. At each lecture there will be a band, usually one of the famous Yorkshire bands, with either a choir or two or three vocalists. Every Yorkshireman loves music, and these occasions ought therefore to be doubly attractive, since those who are not qualified to criticise the lectures will at least consider themselves entitled to criticise the music. We hope the admirable example of Sheffield will be widely followed. It would, indeed, be well if similar lectures were arranged in every great town, for upon no subject of universal moment is there greater need for the dissemination of knowledge than the public health.

### SOBRIETY BY TAXATION.

THERE are many means by which sobriety can be achieved, or at least sought. One is by an appeal to the pocket. This is the system which has been adopted in Denmark since 1917, and it has worked remarkably well if we are to judge from statistics recently published as to the incidence of delirium tremens since 1917, in a hospital in Copenhagen. It appears that in 1917 such heavy taxation was imposed on spirits that they now cost ten times as much as formerly. There are several ways by which the sobriety of a community may be gauged, but there is probably none so effective as the calculation of the number of cases of delirium tremens treated in hospital from year to year. Doubts there may be as to the diagnosis of chronic alcoholism, but even artless maiden ladies usually know delirium tremens when they see it. The number of cases of delirium tremens treated in a hospital in Copenhagen in the period 1903-1916 ranged from a minimum of 249 in 1915 to a maximum of 447 in 1907. The annual average for this period was 321 cases. In the period 1918-1922 this annual average had fallen to 15—that is to say, 95 per cent. of the anticipated number of cases of delirium tremens did not occur. Could the remaining 5 per cent. also vanish ? It is probable that in every community there is a residuum of topers whom it is impossible to sober. If Prohibition is substituted for heavy taxation, there are always the alternatives of the private still, smuggling, and debauchery with methylated spirits, petroleum, ether and the like. But an achievement which comes within 95 per cent. of perfection is one of which the Danish nation may well be proud.

### LIFE ASSURANCE AND SURGERY.

SINCE death sometimes follows an operation, there is an obvious connexion between life assurance and surgery, but that is not quite the connexion which has moved a great insurance company to extend the benefits it offers to its assured. It has just announced that in the case of endowment assurances for £1,000 and upwards it will reimburse, more or less, the cost of a surgical operation. For each £1,000 of assurance it will advance a maximum of sixty guineas for surgeon's fees and thirty guineas for nurse's fees, charging the amount upon the policy, but the offer will not apply when the conjoined fees do not exceed £25. To the well-to-do a surgical operation causes no financial inconvenience, while people of very small means can be attended to in hospitals. The middle-class man, who depends upon his work for his livelihood, is at a grave disadvantage, and a provision of this kind will in many cases be a very present help. Since, however, most life assurance policies are probably for smaller sums than £1,000, it is to be hoped that experience may show the feasibility of reducing the limit to £500. Meanwhile we may well be glad to see life insurance thus assisting in the means to health.

### THE PREVENTION OF GONORRHŒAL OPHTHALMIA.

IT has been calculated that in certain countries about 20 per cent. of all cases of blindness are due to infection at birth of infants' eyes with the gonococcus. A recent report from an institute for the blind in Denmark shows how remarkably effective Credé's method for preventing gonorrhœal ophthalmia may be when adopted wholesale. In 1900 it became obligatory, in Denmark, for every midwife to instil a 1 in 150 solution of silver nitrate into the eyes of every child at birth, irrespective of the social and moral standing of the parents. This universal adoption of Credé's method has already left its mark on the incidence of blindness in Denmark. In the period 1882-1902 the institute referred to admitted 365 blind persons for training. In 50, or 13.7 per cent., of these cases the blindness was due to infection of the eyes with the gonococcus. In the period 1903-1923 the same institute admitted 372 blind persons, only 33 of whom (8.8 per cent.) had become blind on account of gonorrhœa. Since 1904 the average number of cases of gonorrhœal blindness admitted to this institute has been one case every other year. These are very remarkable figures, and they certainly point to the wisdom of making Credé's method compulsory; as long as it is not compulsory the position of the physician or midwife who insists on taking this precaution is most invidious. He or she is liable to be regarded as a distrustful, suspicious person, scenting mischief where there is none. Only by making Credé's method universally compulsory is every case of infection of the infant's eyes during birth likely to be aborted.

### THE WORLD'S RADIUM.

RADIUM and its medical applications are always surrounded, in popular estimation, by a certain amount of mystery. The very rarity of the element itself assures this. Certainly it is not generally realised how much more extensive our sources of radium have become within the last few years. The great changes occurred in 1913 with the discovery of new sources of uranium in the Belgian Congo, and particularly with the detection of the Kasolo deposit in 1915 by a British prospector, Sharp. Naturally development was held up by the War, and until 1919 Colorado and Utah still stood as the chief sources of radium. Up to that time about 160 grammes of the bromide had been produced. The new Belgian deposits proved, however, to be proportionately about 30 times richer in radium and so easy to work that the American producers have, in face of the competition, largely closed down. From the Congo the raw material is brought to Antwerp, where extraction is carried on by a process which is still essentially the same as that originally invented by Madame Curie. At present the Belgian company offers radium at about £4 per milligram, and the price will probably be still further reduced.

### DISADVANTAGES OF ALUMINIUM PAINT]

WE see so frequently the application of bronze and aluminium paints to water pipes and radiators, and the process is so rapid and therefore so appealing to institutional decorators, that any possible coincident disadvantages should certainly be known. A series of careful experiments detailed by the *Chemical Trade Journal* bring out strong evidence that radiators treated in this way markedly lose their heating efficiency. It is said that the heat radiated from an aluminium painted radiator surface is less than one third that provided by one painted with non metallic paint, enamelled or simply allowed to rust. This observation has, of course, another application. If we do not want too much heat to be transmitted through, and therefore lost from, a metallic surface, it is a good plan to cover it with the paints in question. The experimenters find that their application is a very effective means of reducing the amount of heat transmitted through any thin material, and, as a rule, radiators transmit far too much heat.

### CHILD LIFE IN THE DOMINIONS.

THE average infantile death-rate for the Dominion of New Zealand—always remarkably small—has, according to the Report of the Director-General, been yet further reduced to just under 4.2 per cent., which is the lowest on record. While New Zealand is congratulating itself, the Transvaal is lamenting its high infantile mortality which Dr. Leipoldt—a former member of the editorial staff of the HOSPITAL AND HEALTH REVIEW and late Medical Inspector of Schools for the Province—attributes to the ignorance of the parents as to their responsibilities and their physical obligations to their offspring. Propaganda work, in his opinion, is urgently called for. Since in New Zealand the strenuous propaganda campaign is quite evidently having a stimulating effect in matters of child welfare, it is probable that the same excellent results would follow in the Transvaal. There, however, the population is unfortunately still ignorant of elementary hygiene, and little in the way of cooperation with the medical authorities is forthcoming. Health lectures to parents have attained a certain popularity but, as Dr. Leipoldt suggests, the School Clinic is perhaps the most valuable means of breaking down old prejudices and supplying a concrete object lesson in the laws of health. Where there is so little knowledge among the parents, the proportion of defective children is bound to be high, though medical inspection has already materially reduced the percentage of defectives in schools. Ultimately Dr. Leipoldt thinks this should not exceed a percentage of 15 in primary and 5 to 8 in secondary schools. "Natural advantages are with us; we have an admirable climate, our food supplies are commensurate with our needs, and we have by no means touched that depth of abject poverty which accounts for so much defectiveness in European cities." A great deal has been accomplished after eight years of organised medical inspection, and the future looks particularly bright.

### PATIENTS OF THE LAW.

At the recent meeting of the Law Society at Plymouth, Mr. P. H. Edwards, a London solicitor, read an interesting paper suggesting that an institution for providing poor persons with legal assistance should be established on lines similar to those of a voluntary hospital. He called those solicitors who now sometimes take poor persons' cases "panel solicitors without the panel doctor's fee." In his proposed institute a staff of solicitors would be employed, as house surgeons are now in hospitals; the educational side, he hoped would develop on the parallel of the medical schools. The help would be given free, and only out-of-pocket expenses recovered from the "other side" in successful actions. On the practical value of the proposal the Law Society must decide, but it is interesting and heartening to find the voluntary system, which has inspired so many, now taken as a pattern for the Law in the effort to provide for the "casualties" that come to, or short of, the Courts, as accidents and the like now come to, or stand in need of, the hospitals.

### THE MEASUREMENT OF INTELLIGENCE.

Most of us know of some child or other who is particularly intelligent; if we are parents probably the chosen child is our own. If so, it is an entirely excusable failing; but meanwhile we should realise that intelligence is a truly measurable quantity. It is not difficult to apply appropriate tests to any child, and the result is often instructive as well as corrective to our first formed ideas. Remember that, except when dealing with very large groups of population, it is not justifiable to say that the larger their heads the more "clever" the children. The only fair method of estimation is to compare a child's performance, the score it can make in essaying a series of intelligence tests, with that obtained by the average. We cannot here give details of the tests, but anyone professionally connected with child teaching will provide us with such things as counting tests, maze tests, colour selection tests, etc., to suit all ages, and with them the scores the average child should make. Supposing a child of ten years can, according to the standards, perform only as well as an eight year old, then his "mental age" is considered to be eight years and his intelligence quotient is therefore only 80 per cent. The normal child has of course a quotient of 100. The surprising thing about a quotient judged in this way is its remarkable constancy throughout the developing period of life. A child with 80 at six years will generally show exactly the same or only very slightly better figures at twelve years, in spite of most careful and specialised methods of schooling. Some readers may contradict this generalisation and give instances, but the fact remains that, if new tasks be set and no methods learned by heart during the intervening years be allowed to enter into the tests, then the percentage intelligence of the child does not really increase. He may be trained to special things but his power of self-adaptability and initiative remains constant. Such at least is the finding of many observers of thousands of children, and depressing though it may be to those responsible for instructional work, the fact remains, and should be realised when welfare work and improvement schemes are considered.

### THE NATURE OF THIRST.

To most of us thirst must seem to be relatively simple of explanation—the natural signal that the body as a whole requires liquid. It is clear, however, from a discussion of the point in the *Journal of the American Medical Association* that it cannot be thus lightly dismissed. The questions are whether the sensation of thirst is general to the whole body and evoked only by loss of water from the tissues or whether it is simply limited to the mucous membranes of the mouth and throat. If the latter be the case, then relief should be possible by local treatment alone, and, as far as mild thirst is concerned, experiment seems to prove that it is. Certainly we all know that the thirst produced by drying of the throat in prolonged singing or speaking, constant mouth breathing and so on, can usually be relieved by moistening the fauces without actually swallowing the water. But a more striking experiment can be made in animals. If drugs, such as pilo-carpine, which stimulates the flow of saliva, be injected, then for some time the animal, though previously crying for water will show no signs at all of thirst. In short, the natural sensation has been locally suppressed although no water has entered the animal's system meanwhile. On the other hand it is impossible to obliterate by any such means the severe thirst which accompanies certain diseases causing constant and rapid loss of water. Severe thirst is evidently a general sensation and relieved by nothing but the actual drinking of much water. It is seen, therefore, that the question of the exact origin of the sensation is still open, but thirst being such a commonplace, the very fact of this uncertainty seems to make the point worth notice.

